# Curriculum vitae

**Published:** 2012

**Figure:**
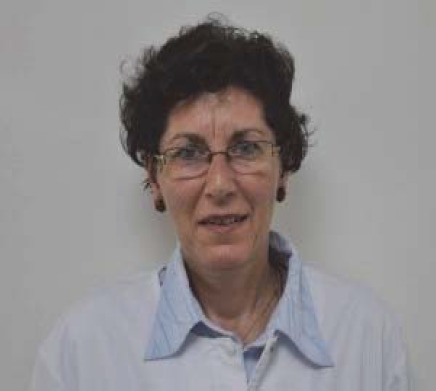


**Anca Roxana Lupu, MD PhD**

**CURRENT POSITION:**

Manager of Coltea Clinical Hospital Bucharest

Professor - “Carol Davila” University of Medicine and Pharmacy-Bucharest from 2005

Supervisor PhD from 2007

Head of Hematology Department -Coltea Clinical Hospital Bucharest

President of the Romanian Society of Hematology

Vice-president of the Romanian Society Haemostasis and Thrombosis

Member of the Council of Professors “Carol Davila” University of Medicine and Pharmacy -Bucharest

Member of the College of Physicians in Bucharest

Member of the General Assembly of the College of Physicians of Romania

Member of advisory expert committees of the Ministry of Health and National Health Insurance House

**EDUCATION:**

Graduated from “Carol Davila” University of Medicine and Pharmacy as Ensign

**POSTGRADUATE TRAINING**

1993 to present-MD Internal Medicine

1998 to present-MD Hematology Clinic

 TEACHING TRAINING;

2005 to present Professor -“Carol Davila” University of Medicine and Pharmacy Bucharest.

**POSTDOCTORAL COURSE**

Project MEDAS - “Training physicians and nurses in management and use of new technologies” - POSDRU/63/3.2/S/20264

**LONG-TERM EXPERT SCIENTIFIC COUNCIL** “To support postdoctoral research in the field of reconstructive surgery transplantation (transplantation Postdoc)” POSDRU/89/1.5/S/ 64153

Doctor of “Carol Davila” University of Medicine and Pharmacy, Bucharest-1996

Membership in scientific societies national and international

Member of scientific teams in specialized medical publications

Peer reviewer of some medical publications

Manual, book chapters and other -15 in total

SUPERVISOR PhD:

1. PhD scholarship, the thesis defended-1

2. PhD scholarship 1

3. PhD extramural toll 10

4. PhD extramural toll - foreign 1

Joined as referenced in numerous thesis committees support “Carol Davila” University of Medicine and Pharmacy, Bucharest, Timisoara, Iasi, Sibiu, Craiova, Constanta.

